# Glyphosate and Glufosinate Residues in Honey and Other Hive Products

**DOI:** 10.3390/foods12061155

**Published:** 2023-03-09

**Authors:** Giulia Rampazzo, Teresa Gazzotti, Elisa Zironi, Giampiero Pagliuca

**Affiliations:** 1 Department of Veterinary Medical Science, Alma Mater Studiorum University of Bologna, Via Tolara di Sopra 50, Ozzano dell’Emilia, 40064 Bologna, Italy; 2 Health Sciences and Technologies-Interdepartmental Centre for Industrial Research (CIRI-SDV), University of Bologna, Ozzano dell’Emilia, 40064 Bologna, Italy

**Keywords:** glyphosate, glufosinate, AMPA, honey, hive products, contaminants, analytical methods

## Abstract

Hive products have numerous beneficial properties; however, the hive’s health is affected by the surrounding environment. The widespread use of herbicides in agriculture, such as glyphosate and glufosinate, has raised alarm among consumers, beekeepers, and environmentalists due to their potential to harm bees and humans through the consumption of bee products. This review aims to provide a comprehensive overview of the presence of glyphosate, glufosinate, and their metabolites in hive products, collecting and comparing available data from peer-reviewed research and surveys conducted across several countries. Moreover, it analyzes and discusses the potential impacts of these substances on human and bee health, analytical aspects, and recent regulatory developments. The data has revealed that these substances can be present in the different matrices tested, but the concentrations found are usually lower than the maximum residue limits set. However, the use of different methodologies with non-uniform analytical performances, together with an incomplete search for regulated analytes, leads to heterogeneity and makes comparisons challenging. In addition to the completion of studies on the toxicology of herbicide active ingredients, further monitoring actions are necessary, harmonizing analytical methodologies and data management procedures.

## 1. Introduction

A beehive has the potential to accumulate environmental contaminants collected by bees from the surrounding land. In fact, in each hive, several thousand foraging bees visit a thousand flowers each day, resulting in at least a few million interactions daily. Furthermore, the area subject to bee activity has been estimated to be around 7 km^2^ [1]. Bees not only gather nectar from flowers and pollen and propolis from buds, but they also drink water from the environment and collect suspended particles that settle on plants [2].

The most common environmental contaminants detected in bee products are heavy metals (arsenic, cadmium, mercury, and lead) and certain pesticides (biphenthrin, triadimefon, lambda-cyhalothrin, and chlorpyrifos) [3].

Among plant protection products, herbicides such as glyphosate (Gly) and glufosinate (Glu) are currently a hot topic [4]. In the late 1990s, the introduction of genetically modified crops resistant to these broad-spectrum herbicides led to a significant increase in their usage, with important economic implications [5]. The global market for glyphosate was estimated at $7.6 billion in 2020 and is expected to reach $9.3 billion by 2027. For glufosinate, estimates are $2 billion in 2020, with a projected increase to $3.5 billion by 2027 [6].

The presence of these substances and their metabolites in the environment poses a risk to bees themselves and can also lead to the contamination of hive products.

In recent years, concern about the effects of these substances on both human health and the environment has increased worldwide. In Europe, the two ‘calls for action’ of the Green Deal’s Farm to Fork and Biodiversity strategies aim to reduce the use and risk of pesticides as well as reverse the decline of pollinating insects [7].

Given the controversial evidence regarding the potential health effects of the herbicides under consideration and recognizing that bees and their products are known indicators of environmental poisoning, data on the contamination of these products is still scarce.

To the best of our knowledge, this is the first systematic review that collects and analyzes available information on the contamination of bee products by Gly, Glu, and their metabolites, considering not only scientific publications but also data produced by official controls and honey producers themselves. This study critically compares the collected data with reported analytical performance and legal limits established in various countries, providing a comprehensive, wide-ranging, and up-to-date overview. Furthermore, it highlights the remaining gaps about data collection and evaluation on contamination levels of hive products. Additionally, the review discusses the methods used, the evolution of analytical techniques, and their performances.

## 2. Methods

A literature search was conducted using various electronic bibliographic databases (e.g., Scopus, PubMed, and Google Scholar) using different keywords such as glyphosate, AMPA (aminomethylphosphonic acid), glufosinate, herbicides, and hive products. The search was further refined by adding honey, beebread, wax, and pollen to the keywords.

Currently, limited information is available on the presence of Gly, AMPA, and Glu in bee products. To have a more complete overview, we consulted institutional databases, reports, and monitoring such as the European Pesticides Database, EFSA’s annual reports on pesticide residues in food, the New Zealand National Chemical Residues Programme Report, the FDA Pesticide Residue Monitoring Database, and local beekeeper association monitoring [8].

**Figure 1:**
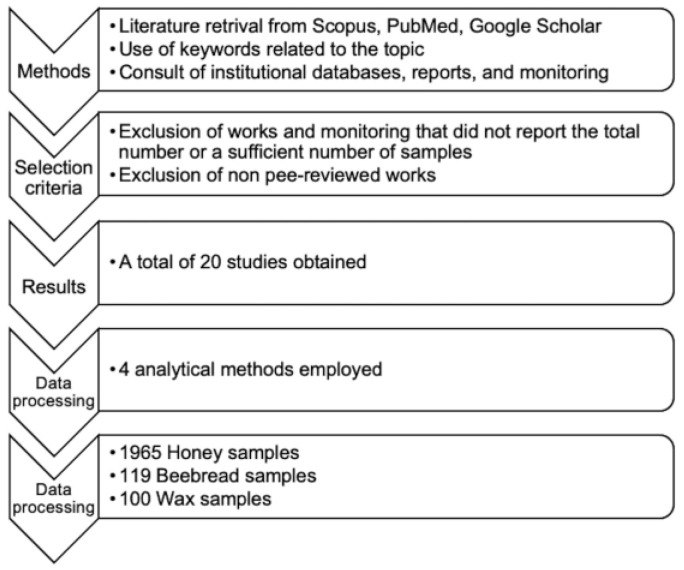
Methodology flowsheet.

This review selected publications based on scientific studies and official surveys examining glyphosate, AMPA, and glufosinate contamination in all hive products and excluded non-peer reviewed works. Additionally, work and monitoring that did not report the total number or a sufficient number of samples (<10) and that did not refer to a validated analytical method were not included. There were no restrictions on the year of publication, author affiliation, or geographic region. The methodological process employed is described in Figure 1.

## 3. Chemical Properties and Mechanism of Action of Glyphosate and Glufosinate

Gly and Glu are both active ingredients in non-selective herbicides that are used to control weeds on agricultural crops. These substances act on key enzymes in the plant’s metabolic pathway, eventually leading to its death.

**Figure 2:**
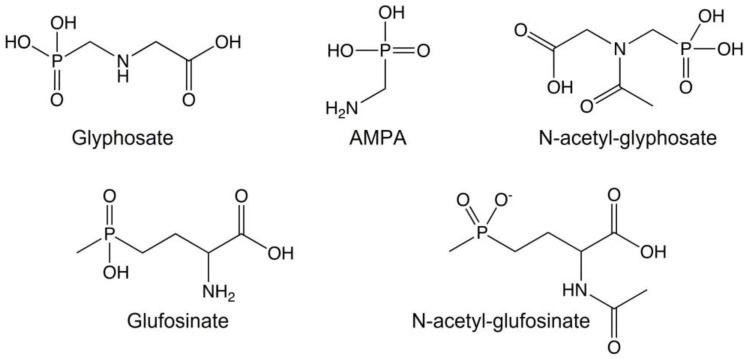
Glyphosate, glufosinate and metabolites chemical structures.

Chemically, Gly and Glu belong to the organophosphate category (Figure 2); however, their mechanism of action is different from that of the organophosphate anticholinesterases, and, as a result, their potential effects on human health may be different.

Gly and Glu are anionic polar pesticides, and due to their physicochemical properties, they may pose an analytical challenge [9].

Glyphosate [N-(phosphonomethyl) glycine] is a synthetic active ingredient in so-called glyphosate-based herbicides (GlyBHs). Currently, there are hundreds of GlyBHs sold under assorted brands. Glyphosate is less soluble in its acid form, so GlyBHs are formulated using glyphosate salts, including isopropylamine, ammonium, sodium, potassium, and trimethylsulfonium. In addition, GlyBHs contain polar surfactants, including polyoxyethyleneamide (POEA), sulfuric acid, and phosphoric acid, which enhance the herbicidal action of Gly by increasing its solubility in water and promoting its penetration and absorption by the plant [10]. Gly inhibits the 5-enolpyruvylshikimate-3-phosphate synthase (ESPS) in the shikimic acid pathway, which occurs in plants, bacteria, and fungi but not in animals, affecting the biosynthesis of aromatic amino acids, vitamins, and many secondary plant metabolites [11].

Glufosinate, marked worldwide under different commercial brands, is the active ingredient of many non-selective herbicides. It is a racemic mixture of two D and L enantiomers [DL-phosphinothricin or 2-amino-4-(hydroxymethylphosphoryl)butanoic acid], often referred to as glufosinate-ammonium because the ammonium-salt formulations are the most widely used. Glufosinate-based herbicides (GluBHs) contain adjuvants that improve their phytotoxicity, such as ammonium-sulfate and surfactants to enhance wettability and herbicide penetration.

Some species of Streptomyces can produce a tripeptide called bialaphos through fermentation. Upon penetrating the plant, it is hydrolyzed to produce the L-enantiomer of glufosinate (L-phosphinothricin) which is herbicidally active [12]. Once inside the plant cell, Glu acts as a glutamate analogue towards the enzyme glutamine synthetase. This irreversible inhibition of the condensation reaction between glutamate and ammonia causes the latter to accumulate inside the cells. This accumulation leads to the disintegration of the chloroplasts and therefore the death of the plant due to the inhibition of photosynthesis [12].

## 4. Degradation of Glyphosate and Glufosinate

A significant amount of Gly is not taken up by plants and is instead dispersed into the environment, where it undergoes rapid degradation through abiotic and biotic processes such as photolysis, thermolysis, and biodegradation. The degradation time (DT50) can vary widely depending on environmental conditions, with soil studies reporting DT50 values ranging from 1 to 130 days [13]. The main Gly metabolite is AMPA, which is more persistent and causes secondary contamination in the environment [14]. AMPA is often detected in surface water, sediment, and groundwater [15]; soil studies report AMPA DT50 values ranging from 76 to 240 days [13]. Other known Gly metabolites, such as HMPA [(hydroxymethyl)phosphonic acid], N-acetyl-glyphosate [N-acetyl-N-(phosphonomethyl)glycine], and N-acetyl-AMPA [(acetamidomethyl)phosphonic acid], are currently understudied.

Glu is also rapidly degraded by plants and soil bacteria through oxidation, transamination, and acetylation reactions [12]. Laboratory studies on the rate of degradation in soil have reported DT50 values from 6 to 11 days [16]. Its main metabolite is MPP [3-(hydroxy(methyl)phosphinoyl) propionic acid], which may undergo further degradation to MPA (2-methylphosphinico-acetic acid) and N-acetyl-glufosinate. MPP and MPA are moderately persistent, with soil DT50 values of 13–20 days and 17–18 days, respectively [16].

## 5. Glyphosate and Glufosinate Effects on Honeybees’ Health

Recent evidence suggests that honeybee populations are declining or suffering from Colony Collapse Disorder (CCD), a phenomenon in which most worker bees abandon the hive, leaving behind the queen, nurse bees, and larvae. Among the causes of CCD, herbicide use may have adverse effects on bees not only directly but also indirectly by reducing the availability of nectar- and pollen-producing plants [17]. The impact of these polar pesticides on non-target organisms is still unclear and controversial; a recent review examined the effects of Gly on bee mortality through a meta-analysis of articles published between 1945 and 2020 [2]. The analysis showed that Gly and GlyBHs exposure may have toxic, even lethal, effects on bees, including impacts on cognitive abilities and sleep. Both adult bees and larvae were found to be sensitive, and chronic exposure was found to be the most harmful [2]. The IUPAC Pesticides Properties Data Base (PPDP) reports the following Gly ecotoxicity values for honeybees: contact acute LD₅₀ > 100 μg bee⁻^1^; oral acute LD₅₀ 104 μg bee⁻^1^.

Although limited information is available on the toxic effects of Glu, it appears to have minor toxicity due to its rapid degradation in the environment. The PPDP also reports the following Glu-ammonium ecotoxicity values for honeybees: contact acute LD₅₀ > 345 μg bee⁻^1^; oral acute LD₅₀ 600 μg bee⁻^1^. Ecological risk assessments for pesticide registration should consider the potential risks to bees and other pollinating insects from the application of high concentrations of herbicides to crops that attract them. Label use restrictions should be implemented to reduce bee exposure [18,19].

## 6. Regulatory Agencies’ Assessment on Health Effects in Humans and Regulatory Developments

The toxicity of Gly is still a topic of debate. Recently, EFSA proposed an acute reference dose (ARfD) of 500 µg/kg of body weight [20]. Exposure to Gly may cause general human health problems, such as bladder and liver toxicity, severe eye damage, and endocrine issues [21,22]. Over the past few years, there has been significant discussion regarding Gly’s potential carcinogenicity. In 2015, the International Agency for Research on Cancer (IARC) declared Gly as “probably carcinogenic to humans” due to limited evidence of carcinogenicity in humans and sufficient evidence of carcinogenicity in experimental animals [23]. This sparked widespread concern and debate about its safety. In contrast, other regulatory agencies, such as the European Food Safety Authority (EFSA) [20] and the US Environmental Protection Agency (EPA), concluded that Gly is unlikely to pose a cancer risk to humans [24]. In 2017, the EU renewed the license for the use of Gly for five years, with some restrictions on its use; however, the renewal was controversial and opposed by some member states and environmental groups. Supporters of the renewal argue that the chemical is essential for modern farming, and that without it, food production would suffer. Opponents of the renewal maintained that Gly is dangerous and that alternatives should be explored [25]. In December 2022, the EU extended the approval of Gly by one year to allow EFSA sufficient time to conclude its new peer review [26].

Glu can be harmful to humans when ingested or when it comes in contact with the skin. The World Health Organization (WHO) classifies Glu is as a category III compound, meaning that it is “moderately hazardous” and may cause “temporary incapacitation or possible residual injury”. In humans, Glu can cause symptoms such as eye, skin, and lung irritation. Ingestion of Glu can lead to nausea, vomiting, and diarrhea. In some cases, it may also cause tremors, convulsions, and difficulty breathing [27]. According to the IUPAC Pesticides Properties Data Base (PPDP), Glu is a possible toxicant for the kidneys, bladder, blood, and lungs [21]. While Glu was authorized for use in Europe until 2018, its registration was not renewed by the European Commission due to concerns about its toxicity [12,28]; however, Glu continues to be widely used in the United States, South America, and other parts of the world, both on crops and in non-agricultural areas [12].

To protect consumer health, the European Union has set maximum residue limits (MRLs) for pesticides in honey and other apiculture products; however, Regulation (EU) 2018/62 [29] clarifies that MRLs for honey do not apply to other apiculture products due to their different chemical characteristics. The MRL for glyphosate is set at 50 µg/kg according to Commission Regulation (EU) No 2013/293 [30].

For glufosinate [sum of glufosinate isomers, its salts, and its metabolites (MPP and N-acetyl-glufosinate)] Commission Regulation (EU) 2016/1002 set the MRL at 50 µg/kg [31]. Similar limits have been adopted in other countries. In Japan, the MRL for the sum of glyphosate and N-acetyl-glyphosate calculated as glyphosate is set at 50 µg kg for honey (including royal-jelly), with no limits set for other metabolites or for glufosinate [32]. In Australia and New Zealand, the MRL for the sum of glyphosate, N-acetyl-glyphosate, and AMPA, expressed as glyphosate in honey, is 200 µg/kg [33], and no limit has been set for glufosinate. The US and Canada have not established an MRL for glyphosate or glufosinate in honey [34]. Table 1 shows the different MRLs set for Gly and Glu in various countries.

## 7. Analytical Methods for the Determination of Glyphosate, Glufosinate, and Their Metabolites in Honey and Other Hive Products

The determination of Gly, Glu, and their metabolites in honey and other hive products is a challenging task; this is due to their small molecular size and physicochemical properties, such as high polarity, lack of ultraviolet absorption, high solubility in water, low ionization, and low volatility. Furthermore, the high solubility in water and small size of these compounds make selective extraction difficult, resulting in a strong matrix effect [35]. Additionally, the high concentration of sugars can lead to instrument contamination when combined with a non-selective extraction.

Table 2 presents a summary of the main analytical methods used to date for the detection of Gly, AMPA, and Glu in honey, bee bread, and wax, along with their corresponding limits of detection (LOD) and quantification (LOQ). Cases where derivatization techniques were utilized are also included. Upon reviewing the research studies, it is evident that they are all quite recent, having been published within the last five years, except for Guo et al. [35], which dates to 2014. This may be attributed to the increasing attention given to these pesticides as well as new developments in the analytical field that have addressed some of the challenges mentioned previously. The techniques listed in Table 2 are highly diverse and range from extremely rapid enzyme screening methods to sophisticated instrumentation such as HRMS (high-resolution mass spectrometry).

From Table 2, the main methods used for the analysis of these pesticides involve the use of liquid chromatography combined with mass spectrometry (MS) and, in only one case, coupled with a fluorescence detector (FLD) [41].

Only in three studies was Gly detected in honey by enzyme-linked immunosorbent assay (ELISA). However, the type of kit used (Abraxis Glyphosate Plate Assay Kit 500086) was designed to analyze glyphosate in water (groundwater, surface water, well water), therefore it had to be adapted to different matrices, including honey [35,44,45].

As far as the chromatographic approach is concerned, a few years ago the use of traditional reversed-phase liquid chromatography did not provide optimal retention of small polar analytes and often required the use of derivatization. Derivatization analysis permits good separation even on reverse phase columns, but presents problems related to contamination of both columns and detectors by the derivatizing agents’ byproducts. The most commonly used derivatizing agent is FMOC-Cl (9-fluorenylmethoxycarbonyl chloride) [34,37]; an author proposes dansyl cloride (5-(dimethylamino) naphthalene-1-sulfonyl chloride) as an alternative, reporting less interference [38]. Making these pesticides fluorescent is necessary when using fluorescence detectors. In the work of de Souza et al., this step was performed by post-column derivatization with o-phthalaldehyde (OPA) [41].

More recently, analysis in liquid chromatography without derivatization has been made possible by the development of columns based on different approaches, often using multiple retention mechanisms such as hydrophilic interaction liquid chromatography (HILIC), weak anion exchange (WAX), weak cation exchange (WCX), and porous graphitic carbon stationary phases. However, these approaches have only recently been improved, resulting in adequate robustness for routine analytical use.

Ion chromatography (IC), a High-Performance Liquid Chromatography of small charged solutes, is another potential option, although it is not yet widely used.

As shown in Table 2, the use of tandem mass spectrometry (MS/MS) systems coupled with chromatography is prevalent among the detectors used for the determination of herbicides in hive products. These instruments can provide high performance, meeting all the sensitivity and selectivity requirements needed for this type of analysis. The use of high-resolution mass spectrometry (HRMS) can further enhance these performances.

The “Quick Polar Pesticides Method” (QuPPE), developed and regularly updated by the EU Reference Laboratories for Residues of Pesticide, enables the analysis of a selection of highly polar pesticides (including Gly, Glu, and various metabolites) in foods of plant origin and honey. This method involves extraction with acidified methanol and the use of LC- or IC-MS/MS techniques to analyze multiple combinations of pesticides simultaneously [49].

The quality of the exposure assessment is largely dependent on the quantity and accuracy of the data available and how it is utilized.

The risk analysis is frequently challenged by datasets composed of large amounts of left-censored values, which are difficult to analyze statistically. In particular, this refers to chemical contaminant data reported to be below the limit of detection (LOD) or limit of quantification (LOQ). Left-censoring indicates that there is a limit of contamination below which values cannot be accurately determined. The substitution method should be used to deal with left-censored values, which involves replacing non-detects with either zero or LOD or LOQ depending on the lower and upper bound scenarios [50]. This approach was used in only one study examining glyphosate contamination in honey [44].

As reported in Table 2, the values of LOD and LOQ can vary greatly depending on the method used: ELISA methods show LOQs ranging from 0.4 to 15 µg/kg, LC-MS/MS methods reported limits between 1 and 50 µg/kg, and IC-HRMS range from 5 to 9.26 µg/kg.

The lack of reporting of the LOD in the methods being examined, as well as the LOQ values being either very low or corresponding to the MRL set by the EU, affects the values of left-censored and quantifiable samples from the different monitoring shown in Table 3.

## 8. Reported Levels of Herbicide Contamination in Hive Products

In recent years, thanks to the development of specific analytical methods, some studies and monitoring have been conducted on the presence of herbicide residues (in particular Gly, AMPA, and Glu) in hive products.

Some of these investigations looked at factors that may influence the level of contamination in these matrices beyond the amount of herbicide used and the frequency of its application. The environmental conditions can also play a role in the herbicide contamination of honey; for example, rainfall can wash herbicides off plants and into nearby water sources, where they can eventually be taken up by bees. Additionally, wind can carry herbicides further afield, potentially increasing their presence in honey.

The results of a survey conducted in New Zealand in 2017/2018 [51] suggest that inadvertent exposure of honeybees to Gly from its approved use in agriculture and on pastures is the most likely source of its residues in honey. Therefore, beekeepers have no way to prevent bees from gathering nectar and pollen from plants sprayed with this herbicide. Clover and pasture, or multifloral/blend honeys, were found to have a higher frequency of Gly residues.

The research results from northern Italy indicate that Gly is present in beehives and their products throughout the year, with concentrations varying by season, and its presence appears to be increasing over time. Additionally, the herbicide was detected in pollen collected at an elevation of 850 m above sea level. This discovery challenges the assumption that glyphosate in hives is due to drift from nearby agricultural areas and proves that even remote areas are subject to contamination [8]. On the other hand, AMPA is rarely found in hives and hive products, indicating that the contaminant is not being collected from water, where the concentration of the metabolite is much higher than the parent molecule [8].

The results of monitoring for Gly, AMPA, and Glu in honey, beebread, and beeswax samples are summarized in Table 3, which includes data from scientific articles in the literature, national monitoring by control authorities [51,55,56,57], and monitoring by beekeepers’ associations [8], totaling 20 studies.

Table 3 presents the total number of samples analyzed per matrix, the number and percentage of left-censored and quantifiable samples, along with their relative contamination range, mean, and median (when indicated). The outcomes are listed in chronological order, starting with the most recent results.

Regarding honey, it appears to be the most extensively monitored matrix, with 20 studies conducted. A total of 1965 honey samples were analyzed, and all were investigated for the presence of Gly. It was found in 625 samples (32% of all samples), with contamination ranging from 2.0 µg/kg up to 5500 µg/kg, with maximum levels of 3500 µg/kg in Pakistan [53] and 5500 µg/kg in Europe [48]. Nevertheless, there is very little consistent data available, so drawing conclusions about the actual contamination and factors that may influence the presence of this pesticide is extremely risky. The reviewed documents rarely compare contamination levels with MRLs set by different countries. A comparison of the few available mean or median values shows that, in most cases, the reported concentration is below the MRL [34,37,39,46,48]. However, in other cases [52,53], it is well above, with averages 20 or 40 times higher than any limit.

In nine studies, concentrations of AMPA ranging from 1.9 µg/kg to 100 µg/kg were detected in 208 (44%) of the 471 honey samples tested.

It is noteworthy that Thompson et al. [34] found the highest rate of samples testing positive for both Gly (99%) and AMPA (99%). A similar figure was recorded in a study conducted in Switzerland [46], where the percentage of quantifiable samples for Gly was 94%. This high rate of quantifiable samples may be due to the low declared LOQ value of 1 µg/kg.

If monitoring and scientific research related to Gly contamination in honey are limited, those related to Glu are even rarer; in fact, Glu was only considered in four studies, with a total of 322 analyzed samples. It was found in only one study by Thompson et al. in 2019 [34] in 125 samples (39%) of Canadian honey, with contamination ranging from 1 µg/kg up to 33 µg/kg. The scarcity of data on Glu contamination in honey could be due to several factors, such as its lower use compared with Gly, its rapid degradation in soil, and the non-renewal of registration in Europe since 2018. Furthermore, data on the sum of Glu and its main metabolites, as required by the EU MRL, has never been reported. In particular, the presence of MPP was never investigated in honey. Regarding N-acetyl glyphosate and N-acetyl glufosinate, only a single sample of honey has been reported in the EU Pesticides Database with unquantifiable values [52].

**Figure 3:**
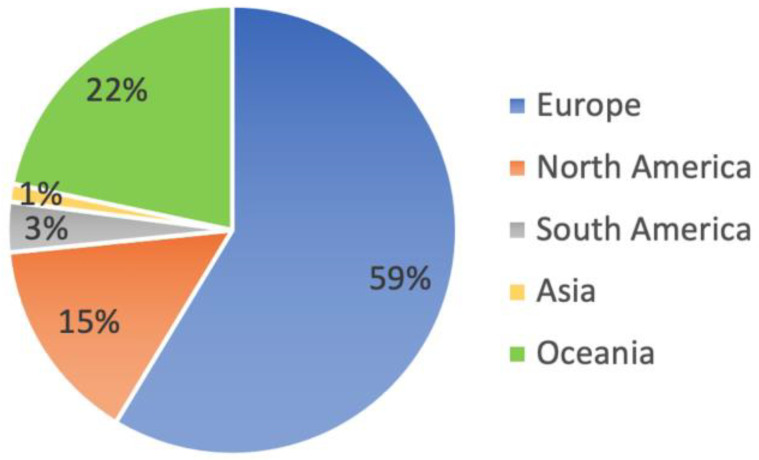
Percentage distribution of the number of samples analysed for glyphosate residues in honey samples across continents.

Figure 3 illustrates the honey samples that were tested for Gly in various parts of the world, with the majority (59%) being tested in Europe, followed by Oceania (22%), and North America (15%). The European results are quite varied, with some surveys [52] showing mean and median values that are much higher than the EU MRL, while others have no quantifiable samples [43,54,55,56,57]. In Italy [8,38,43,54], where more research has been conducted on these analytes, the data is very inconsistent. As already mentioned, these variabilities could be due to different analytical performances.

Among other hive products, two studies analyzed bee-bread samples and found that 72% (119 samples) of the 165 samples tested were contaminated with Gly, with concentrations ranging from 10 to 700 µg/kg. AMPA was measured in beebread only by El Agreibi et al., with contamination levels ranging from 10 µg/kg to 250 µg/kg and a quantifiable sample percentage of 26% [39].

This research was the only one to analyze the presence of Gly and AMPA in beeswax. Out of 100 samples, 32% tested positive for Gly, with levels ranging from 10 to 320 µg/kg. No samples were found to contain quantifiable amounts of AMPA [39]. The results of a recent study [58] showed that all 120 pollen samples tested contained Gly, with levels ranging from 5.07 µg/kg to 7.29 µg/kg; however, these findings were not included in those processed because they were reported in a preliminary report that had not undergone peer review. No other pollen contamination data were found.

No studies were found on the levels of Gly or Glu and their metabolites in other hive products, such as royal jelly or propolis. Additionally, the presence of Glu was not investigated in any of the matrices reported above.

## 9. Conclusions

Active substances in herbicides can accumulate in the environment and be transferred to bees and their hive products. This could pose a hazard to human health if not monitored and controlled efficiently. Therefore, monitoring activities that provide accurate and comparable data can help to evaluate product safety and quality, and to take preventive measures if necessary.

The raw data, which would have provided a more precise understanding of the degree of contamination and allowed for the identification of samples that did not meet the MRL, was not available in most of the sources reviewed. As a result, the scarcity of data on the contamination levels of the herbicides in question makes an accurate risk assessment difficult. While some studies indicate elevated contamination levels, most authors agree that the risk to consumers is relatively low when hive products are consumed. However, evidence suggests that survey and research programs should continue to conduct monitoring activities.

Additionally, the potential toxicity of individual components of herbicides is not fully understood, and there is limited knowledge of the possible synergistic effects of mixtures of their residues. Moreover, it is noteworthy that the monitoring conducted rarely examined all the regulated substances simultaneously.

Harmonizing analytical performance and data processing is also crucial, and the development of new analytical techniques has improved sensitivity and reproducibility. Representative data are required for an accurate dietary exposure/risk assessment, and proper management of non-quantified (<LOQ) and non-detected (<LOD) samples is crucial. The decision on what value to assign to these results can influence their correct interpretation.

Finally, it would be desirable for the raw data from the various surveys to be made freely available so that it can be reprocessed and reanalyzed. Regulatory bodies, researchers, and beekeepers should continue to work together to monitor and limit the use of herbicides and promote sustainable beekeeping practices to ensure the safety of hive products and the environment.

## Figures and Tables

**Table 1:** MRL for glyphosate and glufosinate in various countries.

Country	Matrix	Analyte	MRL (µg/kg)
EU	Honey and other apiculture products	Glyphosate	50
EU	Honey and other apiculture products	Glufosinate (Sum of glufosinate isomers, its salts, MPP, and N-acetyl-glufosinate)	50
Japan	Honey (Including royal jelly)	Glyphosate (Sum of glyphosate and N-acetyl-glyphosate)	50
Australia	Honey	Glyphosate (Sum of glyphosate, N-acetyl-glyphosate, and AMPA)	200
New Zealand	Honey	Glyphosate (Sum of glyphosate, N-acetyl-glyphosate, and AMPA)	200

**Table 2:** Comparison of analytical methods for the determination of Gly, AMPA, and Glu in honey and hive products.

Ref.	Matrix	Analyte	LOD (µg/kg)	LOQ (µg/kg)	Derivatization	Instrumentation
[36]	Honey	Glyphosate	1	n.r.	FMOC	LC-MS/MS
		AMPA				
[37]	Honey	Glyphosate	3	10	-	LC-MS/MS
	Beebread					
[38]	Honey	Glyphosate	n.r.	25	Dansyl cloride	LC-MS/MS
		AMPA		10		
		Glufosinate		10		
[39]	Honey	Glyphosate AMPA	1	10	-	LC-MS/MS
	Beebread					
	Wax					
[40]	Honey	Glyphosate	n.r.	50	-	LC-MS/MS
	Beebread					
[41]	Honey	Glyphosate	20	40	OPA	HPLC-FLD
		AMPA				
[42]	Honey	Glyphosate	n.r.	9.26	-	IC-HRMS
		AMPA		4.3		
		Glufosinate		5.05		
[43]	Honey	Glyphosate	n.r.	5	-	IC-HRMS
		AMPA		20		
[34]	Honey	Glyphosate	n.r.	1	FMOC	LC-MS/MS
		AMPA				
		Glufosinate				
[44]	Honey	Glyphosate	n.r.	15	-	ELISA
[45]	Honey	Glyphosate	0.075	0.4	-	ELISA
[46]	Honey	Glyphosate	n.r.	1	-	LC-MS/MS
		AMPA		2.5		
[47]	Honey	Glyphosate	n.r.	16	-	LC-MS/MS
[48]	Honey	Glyphosate	n.r.	50	-	LC-MS/MS
[35]	Honey	Glyphosate	n.r.	15	-	ELISA

n.r., not reported.

**Table 3:** Comparison of data on Gly, AMPA, and Glu in honey and other hive products reported in different studies (range, mean and median are expressed in µg/kg).

Matrix	Ref.	Origin	Analyte	Total Sample (Number)	LC %	Quantifiable Samples				
						Number	%	Range	Mean	Median
Honey	[36]	Argentina	Gly	30	50	15	50	2.0–27.5	n.r.	n.r.
			AMPA		70	9	30	1.9–18.1	n.r.	n.r.
Honey	[51]	New Zealand	Gly	360	79	78	21	n.r.	n.r.	n.r.
Honey	[37]	Italy	Gly	84	50	42	50	10–34	17.1	n.r.
Honey	[52]	Various EU countries	Gly	115	94	7	6	29–5500	1153	270
			AMPA	35	100	0	-	-	-	-
			N-Acetyl Gly	1	100	0	-	-	-	-
			Glu	14	100	0	-	-	-	-
			N-Acetyl Glu	1	100	0	-	-	-	-
Honey	[53]	Pakistan	Gly	25	80	5	20	440–3500	2004	n.r.
Honey	[39]	Belgium	Gly	10	90	1	10	11	11	11
			AMPA		100	0	0	-	-	-
Honey	[54]	Italy	Gly	98	100	0	0	-	-	-
			AMPA		100	0	0	-	-	-
			Glu		100	0	0	-	-	-
Honey	[41]	Brazil	Gly	40	63	15	37	40–220	n.r.	70
			AMPA		98	1	2	20–100	-	-
Honey	[55]	EU	Gly	249	93	17	7	n.r.	n.r.	n.r.
Honey	[8]	Italy	Gly	176	50	88	50	10–790	n.r.	n.r.
Honey	[42]	Italy	Gly	10	100	0	0	-	-	-
			AMPA		100	0	0	-	-	-
			Glu		100	0	0	-	-	-
Honey	[43]	Uruguay & EU countries	Gly	32	19	26	81	n.r.	n.r.	n.r.
			AMPA		100	0	0	-	-	-
Honey	[34]	Canada	Gly	200	2	197	98	1–49.8	n.r.	4.9
			AMPA		1	198	99	1–50	n.r.	10.3
			Glu		38	125	62	1–33	n.r.	1.4
Honey	[56]	EU	Gly	157	94	9	6	n.r.	n.r.	n.r.
Honey	[44]	USA (Hawaii)	Gly	59	73	16	27	15–342	33.5 LB 118.3 UB	n.r.
Honey	[46]	Switzerland	Gly	16	6	15	94	1–15.9	4.6	3
			AMPA		100	0	0	-	-	-
Honey	[57]	EU	Gly	186	87	24	13	n.r.	n.r.	n.r.
Honey	[48]	Estonia	Gly	33	36	21	64	14–62	44	n.r.
Honey	[47]	USA	Gly	16	44	9	56	n.r.	n.r.	n.r.
Honey	[35]	USA	Gly	69	41	41	59	17–63	n.r.	n.r.
Beebread	[37]	Italy	Gly	84	46	45	54	10–542	n.r.	n.r.
Beebread	[39]	Belgium	Gly	81	9	74	91	10–700	55.5	26
			AMPA		82	21	18	10–250	67.1	44
Wax	[39]	Belgium	Gly	100	68	32	32	10–320	62	36
			AMPA		100	0	0	-	-	-

LC, left-censored; n.r., not reported.

## Data Availability

No new data were created in this study. Data sharing is not applicable to this article.
